# Circular RNA circ0001955 promotes cervical cancer tumorigenesis and metastasis via the miR-188-3p/NCAPG2 axis

**DOI:** 10.1186/s12967-023-04194-4

**Published:** 2023-05-29

**Authors:** Wei Wang, Haixia Luo, Jingjing Chang, Xin Yang, Xiu Zhang, Qingmei Zhang, Yuanxing Li, Yueyang Zhao, Jianbing Liu, Binbin Zou, Min Hao

**Affiliations:** 1 Department of Obstetrics and Gynecology, The Second Hospital of Shanxi Medical University, Taiyuan, 030001 Shanxi China; 2 Pathology Department, School of Medicine, Stanford University, 300 Pasteur Drive, Lane 235, Stanford, CA 94305 USA; 3 School of Applied Science, Taiyuan University of Science and Technology, Taiyuan, 030024 Shanxi China; 4 School of Basic Medical Sciences, Shanxi Medical University, Taiyuan, 030001 Shanxi China; 5 Department of Pathology & Shanxi Key Laboratory of Carcinogenesis and Translational Research on Esophageal Cancer, Shanxi Medical University, Taiyuan, 030000 Shanxi China

**Keywords:** circ0001955, miR-188-3p, cervical squamous cell carcinoma, NCAPG2, AKT-mTOR pathway

## Abstract

**Background:**

Circular RNAs (circRNAs) are known to play a crucial role in a variety of malignancies. However, the precise role of circRNAs in cervical squamous cell carcinoma (CSCC) remains largely unknown.

**Methods:**

The expression of circ0001955 was determined by real-time quantitative PCR and fluorescence in situ hybridization. To examine the effects of circ0001955 on CSCC metastasis and growth, functional experiments were conducted in vitro and in vivo. Mechanistically, nucleocytoplasmic separation, dual luciferase reporter assay, RNA antisense purification experiments, and rescue experiments were performed to confirm the interaction between circ0001955, miR-188-3p, and NCAPG2 in CSCC.

**Results:**

Here, we demonstrated that a circRNA derived from the CSNK1G1 gene (circ0001955) is significantly upregulated in CSCC. The overexpression of circ0001955 promotes tumor proliferation and metastasis, whereas the knockdown of circ0001955 exerts the opposite effects. Mechanistically, circ0001955 competitively binds miR-188-3p and prevents miR-188-3p from reducing the levels of NCAPG2, activating the AKT/mTOR signaling pathway to induce epithelial mesenchymal transformation. Notably, the application of an inhibitor of mTOR significantly antagonized circ0001955-mediated CSCC tumorigenesis.

**Conclusion:**

circ0001955 promotes CSCC tumorigenesis and metastasis via the miR-188-3p/NCAPG2 axis which would provide an opportunity to search new therapeutic targets for CSCC.

**Graphical Abstract:**

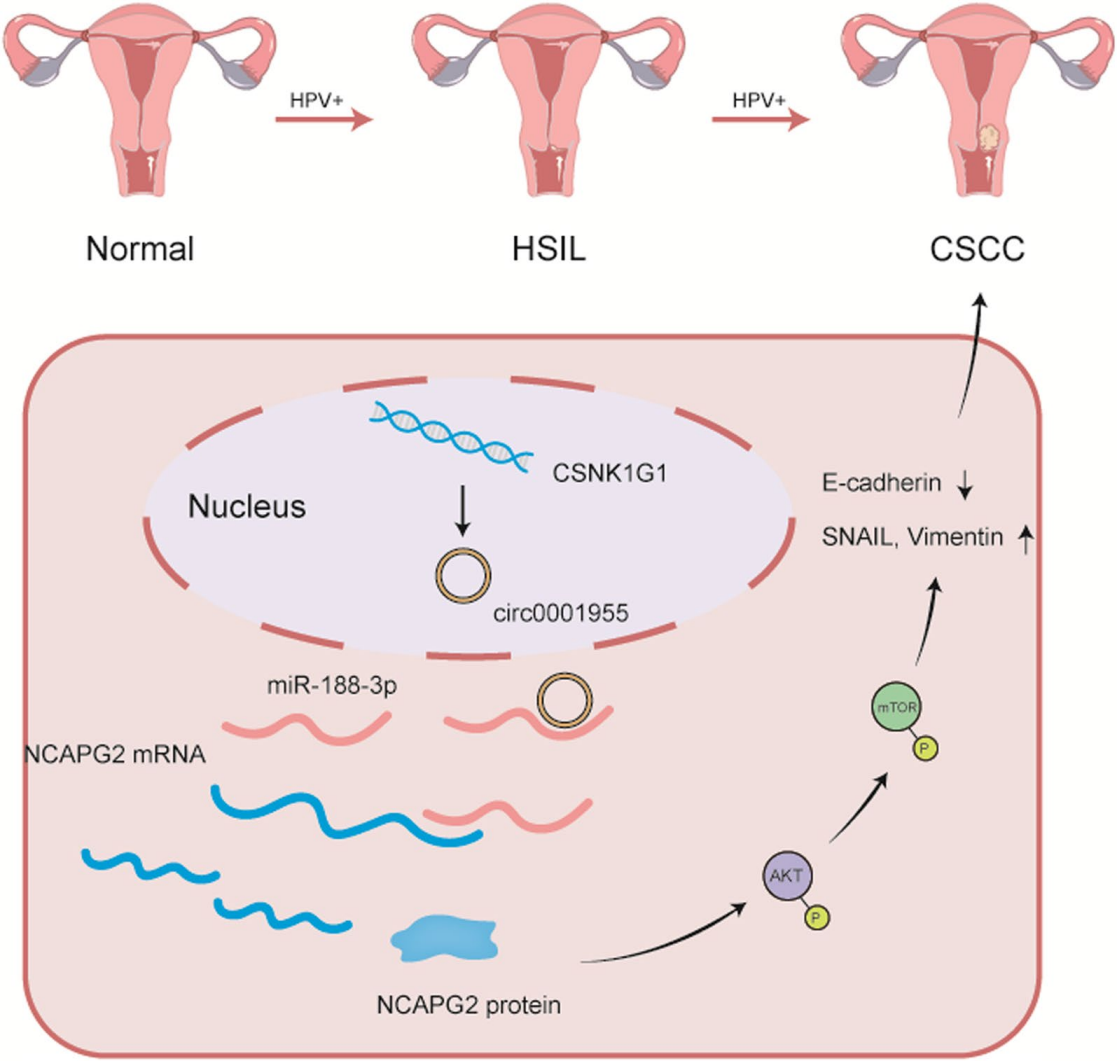

**Supplementary Information:**

The online version contains supplementary material available at 10.1186/s12967-023-04194-4.

## Introduction

Cervical cancer was the fourth most common cancer among women worldwide in 2020, with 604,127 new cases and 341,831 related deaths [1]. Cervical squamous cell carcinoma (CSCC) is one of the most common subtypes of cervical cancer, accounting for 80–85% of all cases [2]. Cervical carcinogenesis has been described as a sequence of steps based on histopathological classifications, from high-risk human papillomavirus (HR-HPV, mostly HPV16) infection, low-grade squamous intraepithelial lesion (LSIL), high-grade squamous intraepithelial lesion (HSIL), and finally invasive cervical cancer [3]. Surgery and radiotherapy are currently the main methods of treating CSCC, although their therapeutic effects are not ideal for recurrent or late-stage disease [4, 5]. Therefore, it is imperative to discover novel molecular targets for the treatment of CSCC patients.

Circular RNAs (circRNAs) are a new type of noncoding RNA that have a continuous, covalently closed loop structure without a 3ʹ-poly A tail or 5ʹ-cap structures [6, 7]. CircRNAs exhibit tissue-specific expression profiles and different levels of expression in varying environments, featuring stable structure and resistance to RNase R treatment compared to linear RNAs [8, 9]. Growing evidence has suggested that circRNAs are involved in many cellular processes, and it appears that circRNAs can also act as competitive endogenous RNAs (ceRNAs) and competitive bind miRNAs to regulate the translation of downstream target genes [10–12]. For example, circFBXW7 competitive binds multiple miRNAs and inhibits cell proliferation in many forms of cancer [13, 14], and circCLK3 promotes the progression of CSCC by competitive binding miR-320a and increasing the expression of FoxM1 [15]. Nevertheless, the specific mechanisms underlying the capability of dysregulated circRNAs to regulate the progression of CSCC remain largely unknown, and further investigations are urgently needed.

We found a novel CSCC-related circRNA (circ0001955) with tumor-promoting properties using our previous circRNA microarray data from normal cervix, HSIL and CSCC tissues and then examined its function in relation to tumor cell proliferation and metastasis [3]. It has been reported that circ0001955 contributes to the tumorigenesis of gastric cancer by competitive binding miR-758 and regulating ZNF217 expression [16]. Moreover, it has been demonstrated that circ0001955 contributes to the development of colorectal cancer by increasing the expression of MYO6 by competitively targeting miR-455-3p [17]. In the present study, we discovered that circ0001955 promoted CSCC proliferation and metastasis by competitive binding miR-188-3p, resulting in the upregulation of NCAPG2 expression and further activating the AKT/mTOR signaling pathway. Collectively, our results indicate that circ0001955 could be a key driver of CSCC progression and could be a valuable marker and independent prognostic factor.

## Materials and methods

### Specimen collection

CSCC, HSIL tissues and normal cervical tissues were collected from patients who underwent gynecological surgery or colposcopy at the Second Hospital of Shanxi Medical University (Taiyuan, China) between 2019 and 2021. Patients in this study had not received radiotherapy or chemotherapy prior to surgery, and their FIGO (International Federation of Gynecology and Obstetrics) stages ranged from Ia2 to IIa2. All patients underwent radical hysterectomy or cervical conization. Normal cervical tissues were obtained from patients undergoing hysterectomy under nonmalignant circumstances. The tissues were first immediately frozen in liquid nitrogen and then frozen at -80 °C until RNA could be extracted. This study was approved by the Ethical Review Committee of the Second Hospital of Shanxi Medical University. The Declaration of Helsinki was followed in all patient studies.

### Cell culture

The human cervical cancer cell line SiHa (HPV 16 +) and a normal cervical cell line (HcerEpic) were purchased from the American Type Culture Collection (ATCC, Manassas, VA, USA). SiHa and HcerEpic cells were cultured in DMEM (Gibco, Carlsbad, CA, USA) with 10% fetal bovine serum (FBS) (Gibco, Carlsbad, CA, USA) and 1% penicillin/streptomycin (Gibco, Carlsbad, CA, USA). Cells were cultured in a humid atmosphere with 5% CO_2_ at 37 °C.

### RNA and gDNA extraction and cytoplasmic and nuclear RNA isolation

Total RNA was extracted from cells or tissues using TRIzol Reagent (Life Technologies, California, US) according to the manufacturer’s instructions. RNeasy Mini Kits (Qiagen, Hilden, Germany) were used to purify total RNA. gDNA was extracted using the Genome DNA Kit (Solarbio, Beijing, China). The nuclear and cytoplasmic fractions were isolated using the Cytoplasmic and Nuclear RNA Purification kit (Thermo Fisher Scientific, Waltham, MA USA). qRT‒PCR was performed on RNA extracted from the fractions to determine the level of cytoplasmic control transcript (GAPDH) and circ0001955.

### RNase R treatment

The RNAs were incubated with RNase R (Geneseed; Guangzhou, China) for 15 min at 37 °C. Then, total RNA from cells was extracted, and qRT‒PCR was used to determine the stability of circ0001955.

### qRT‒PCR and RT‒PCR

qRT‒PCR and RT‒PCR were performed as described previously [3]. All primers were synthesized by Sangon Biotech (Shanghai, China), and the primer sequences are given in Additional file 1: Table S1. GAPDH or U6 was used as internal normalization for qRT‒PCR experiments in this study. Gel electrophoresis sections were observed under an optical microscope (Leica, DMI6B, Germany). Sanger sequencing of cDNA was performed by Sangon Biotech (Shanghai, China).

### Plasmid construction, RNAi and cell transfection

To overexpress circ0001955, the full-length cDNA of circ0001955 was amplified in SiHa cells and then cloned into an overexpression vector (Public Protein/Plasmid Library, Nanjing, China) containing a front and back circular frame, with a mock vector containing no circ0001955 sequence serving as a control. To knock down circ0001955, three siRNAs targeting the back-splice junction site of circ0001955 and a siRNA-NC were synthesized (Public Protein/Plasmid Library, Nanjing, China). The most effective siRNA, si-circRNA2#, measured by qRT‒PCR was subcloned into the lentivirus vector to construct the sh-circ0001955 vector, while sh-NC was used as the negative control. The lentiviral vector carrying sh-circ0001955 or sh-NC (Hanbio, Shanghai, China) were transiently transfected into SiHa cells. The vectors above were verified by sequencing. MiR-188-3p mimics and inhibitor were purchased from GenePharma (Shanghai, China). miR-NC and inh-NC were used as controls. Transfections were performed using Lipofectamine 3000 (Invitrogen, Carlsbad, USA) according to the manufacturer's instructions.

### Cell proliferation, cell cycle and apoptosis assays

We examined the proliferation activity of SiHa cells using Cell-Light™ EdU DNA Cell Proliferation Kit (Beyotime, Beijing, China) and Cell Counting Kit-8 (Meilunbio, Dalian, China) according to the manufacturer’s instructions. A cloning assay was performed on SiHa cells to determine their cloning capability. Cell cycle analysis was conducted with propidium iodide (PI) staining by flow cytometry (Beckman-Coulter, Hialeah, FL) and analyzed using Modfit software. The Alexa Fluor® 488 Annexin V/Dead Cell Apoptosis Kit (Thermo Fisher Scientific, Waltham, MA USA) was used to identify apoptotic cells.

### Wound healing and invasion assays

At 24 h post-transfection, SiHa cells were seeded in a 6-well plate, scratched with a 10 μL pipette tip in the middle of the wells, and then cultured in serum-free medium. The wound width was evaluated in three independent wound sites per group after 24 h and normalized to the control group. The cell invasion assays were conducted with chambers (8 μm pore size, Corning) and Matrigel (BD Science, USA), while the cell migration assays were conducted with chambers without Matrigel. SiHa cells (2 × 10^4^) were suspended in 200 μL serum-free medium and added to the upper chambers before 500 μL complete medium was added to the bottom chambers and chambers without Matrigel for cell migration. The cells in the upper chambers were removed after 24 h, and the lower chambers were fixed with ethanol, stained with crystal violet, photographed and counted under a microscope (Leica, Wetzlar, Germany).

### Immunohistochemistry (IHC)

For the IHC assay, paraffin sections were incubated with primary antibodies against NCAPG2 (1:100) (Bioss ANTIBODIES, Beijing, China) and Ki67 (1:100) (Abcam, Burlingame, CA, USA) at 4 °C overnight, secondary antibodies at room temperature for 30 min and HRP-labeled streptavidin solution for 30 min and then stained with diaminobenzidine (DAB). IHC staining images were semi-quantified using Image Pro Plus (Media Cybernetics).

### Hematoxylin–eosin (HE) staining

Tissues were immersed in 4% paraformaldehyde, embedded in paraffin, and sectioned into 4 mm thick transverse sections. The sections were then stained with hematoxylin and eosin.

### Dual-luciferase reporter assay

psiCHECK2-circ0001955-mut and psiCHECK2-NCAPG2-mut plasmids were designed and synthesized by GenePharma (Suzhou, China). The Dual-Luciferase Assay System (Promega, Madison, WI, USA) was used to measure luciferase activity as directed by the manufacturer. Then, 5 × 10^3^ cells per well were seeded into 96-well plates. The cells were transfected with WT- or MUT-luciferase reporter vectors and miRNA mimics after 24 h. After 48 h, the relative luciferase activity was examined by the Dual-Luciferase Assay Kit (Promega, Madison, WI, USA) in accordance with the manufacturer’s protocols.

### Fluorescence in situ hybridization (FISH)

FISH assays were conducted to evaluated and recognized the expression and location of circ0001955 and miR-188-3p in cervical tissues and SiHa cells. Briefly, after prehybridization at 55 °C for 2 h, paraffin sections or cell climbing pieces were hybridized with specific Cy3-labeled circ0001955 probes and FITC-labeled miR-188-3p probes (GenePharma, Shanghai, China) at 37 °C overnight and then dyed with DAPI. Slides were photographed with a fluorescence microscope (Leica, Wetzlar, Germany).

### RNA antisense purification (RAP) assays

A circ0001955 biotinylated probe was designed and synthesized by Riobio Technologies (Guangzhou, China). The RNA antisense purification assay kit from BersinBio (Guangzhou, China) was then used to verify the relationship between circ0001955 and miR-188-3p according to the manufacturer's instructions. The antibodies used for the RAP assay included anti-Argonaute2 (AGO2) and GAPDH. Briefly, crosslinked cells were lysed, sonicated and hybridized with the probes for 4 h at 37 °C. Next, the hybridization mixture was incubated with magnetic beads for 1 h. The bound RNAs and proteins were then washed and purified for RNA analysis and western blotting.

### Western blot analysis

Proteins from SiHa cells were extracted using RIPA buffer, separated by 10% SDS‒PAGE, and then electrotransferred onto PVDF membranes (Millipore, Bedford, MA, USA). The membranes were blocked with 5% bovine serum albumin (BSA) and incubated with primary antibodies against NCAPG2 (1:1000) (Bioss ANTIBODIES, Beijing, China), AGO2 (1:1000), AKT (1:1000), phospho-AKT (Ser473) (ABclonal Technology, Wuhan, China), mTOR (1:1000), phospho-mTOR (Ser2448) (Cell Signaling Technology, Beverly, MA, USA) and GAPDH (1:5000) (Santa Cruz Biotechnology, CA, USA) at 4 °C overnight and then incubated with secondary antibodies (1:5000) (Santa Cruz Biotechnology, CA, USA) at room temperature for 1 h. Finally, the bands were examined using enhanced chemiluminescence (ECL) western blotting detection reagents (Beyotime, Beijing, China). The gray intensity of Western-blots data in this manuscript was quantified by ImageJ software (NIH) after normalization to corresponding loading controls.

### Animal experiments

All animal experiments were approved by the Shanxi Medical University Animal Care and Use Committee and complied with the guidelines of the National Institutes of Health. SiHa cells were infected with lentiviruses (Hanbio Co. LTD, Shanghai, China) carrying sh-NC and sh-circ0001955, called LV-NC and LV-circ, respectively, and puromycin was used to select and obtain sh-circ0001955 or sh-NC stably expressed cell lines. In xenograft experiments, 2 × 10^6^ SiHa cells suspended in 50% Martigel Matrix (Corning, USA) were subcutaneously injected into female BALB/c mice (4 weeks). The growth of the tumor was then monitored every 7 days after injection. We measured the tumor size with calipers and calculated tumor volume as length × width^2^ × 0.52. Mice were sacrificed 28 days after injection. Following this, the tumors were removed, weighed, and embedded in paraffin for HE and IHC.

### Statistical analysis

SPSS version 21.0 and GraphPad Prism version 8.0 were used for statistical analysis. Student’s t tests were used to analyze differences between two groups. Survival rates were evaluated using the Kaplan‒Meier method and log-rank test. Pearson correlation was used to determine the correlation between groups. A receiver operating characteristic (ROC) curve was used to assess the diagnostic value. Data are presented as the mean ± standard deviation (SD) across at least three independent experiments. A value of *P* < 0.05 was regarded as statistically significant.

## Results

### Circ0001955 is upregulated in CSCC

**Fig. 1:**
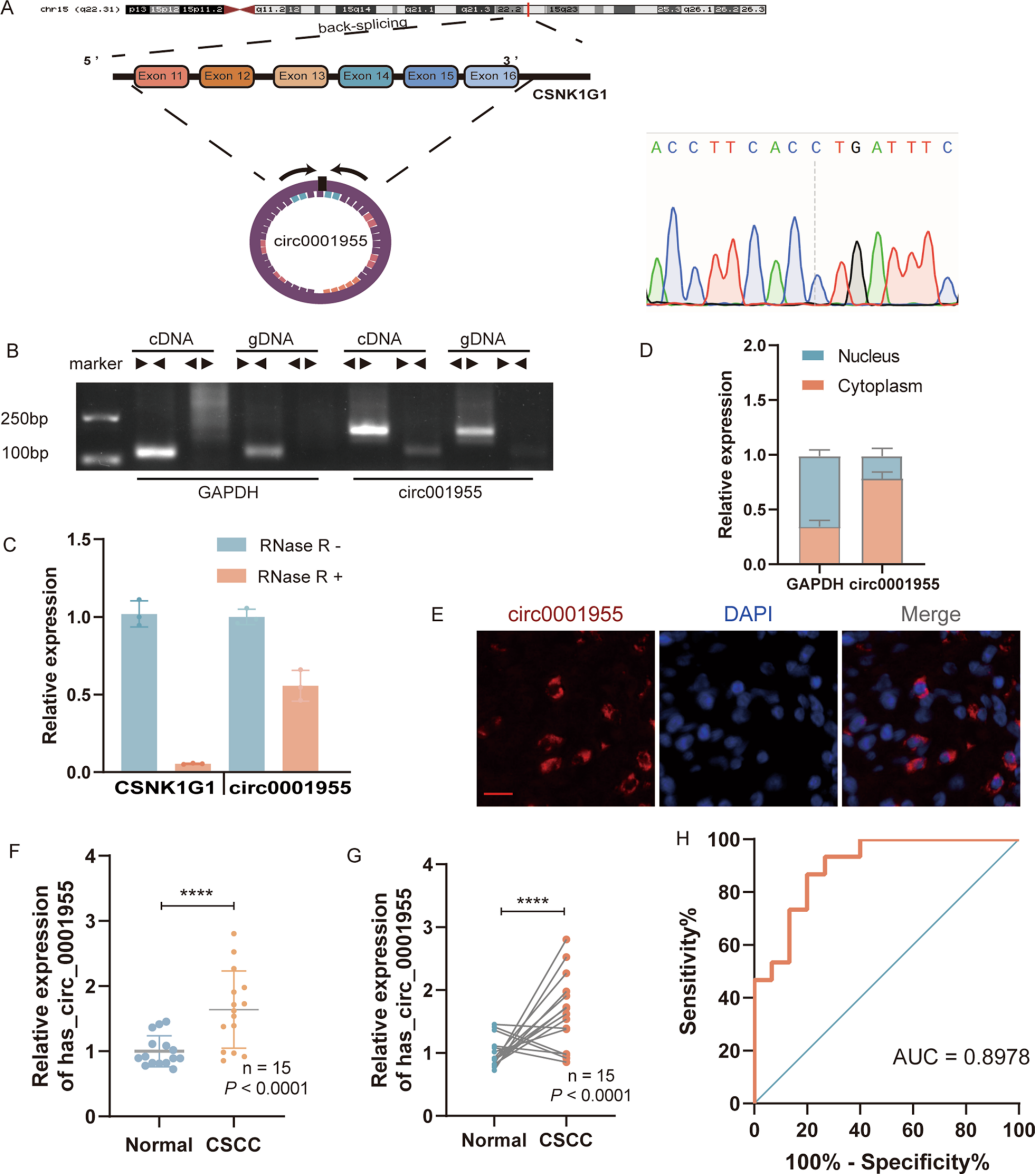
Characterization of circ0001955 in CSCC. **A** The genomic loci of the circ0001955 gene. Circ0001955 is synthesized at the CSNK1G1 gene locus containing exons 11 to 16. The back-splice junction of circ0001955 was identified by Sanger sequencing. **B** PCR analysis for circ0001955 and its linear isoform CSNK1G1 in cDNA and genomic DNA (gDNA). **C** qRT-PCR analysis for the expression of circ0001955 and CSNK1G1 mRNA after treatment with RNase R in SiHa cells. **D** Cytoplasmic and nuclear mRNA fractionation experiments show that circ0001955 is localized mainly in the cytoplasm of SiHa cells. GAPDH was applied as a positive control in the cytoplasm. **E** RNA fluorescence in situ hybridization for circ0001955 in SiHa cells; the junction probe was complementary to the back-splice junction sequence of circ0001955. Nuclei were stained with DAPI. Scale bar, 50 μm. **F** and **G** Expression levels of circ0001955 in CSCC tissues in comparison with matched normal tissues were measured using qRT-PCR. **H** Diagnostic value of circ0001955 for CSCC was evaluated by ROC curve. Each experiment was performed at least three times independently. ****P < 0.0001

Based on our previously published studies [3], we found that circ0001955 was upregulated during the carcinogenic process of the cervix, which is also related to DNA replication and the cell cycle (Additional file 2: Fig. S1 A–D). To confirm the existence of circ0001955, the cDNA of circ0001955 was amplified in SiHa cells and verified by DNA sequencing. We determined that circ0001955 was associated with exons 11 to 16 of the CSNK1G1 gene (chr15:64495280-64508912) and located on chromosome 15q22; circ0001955 was 13,633 nucleotides in length. Then, we designed divergent primers for amplification of the back-spliced junction of circ0001955, and we sequenced this junction by Sanger sequencing (Fig. 1A). We designed divergent and convergent primers that would amplify circ0001955 circular transcripts and CSNK1G1 linear transcripts, respectively, to ensure circ0001955 from head-to-tail splicing in preference to trans-splicing or genomic rearrangements. circ0001955 was only detected in cDNA, therefore eliminating the possibility of it being present in gDNA, whereas convergent primers amplified CSNK1G1 from both cDNA and gDNA (Fig. 1B). Additionally, we found that circ0001955 was more resistant to RNase R digestion than linear CSNK1G1 and GAPDH transcripts (Fig. 1C). qRT‒PCR analysis of nuclear and cytoplasmic circ0001955 was performed to determine its cellular localization. As shown in Fig. 1D, circ0001955 was mostly localized in the cytoplasm of SiHa cells. A similar localization pattern was later confirmed by FISH (Fig. 1E).

Then, the expression of circ0001955 was detected in 15 pairs of CSCC tissues, SiHa cells and HcerEpic cells by qRT‒PCR. In accordance with our microarray data, the results showed that circ0001955 was markedly upregulated in CSCC tissues and cervical cancer cell lines compared with adjacent nontumor tissues and normal cell lines (Fig. 1F, G and Additional file 2: Fig. S1 E). ROC analysis revealed that circ0001955 could sensitively discriminate CSCCs from noncancerous tissues (Fig. 1H).

### Circ0001955 enhances the proliferation, migration and invasion of SiHa cells

**Fig. 2:**
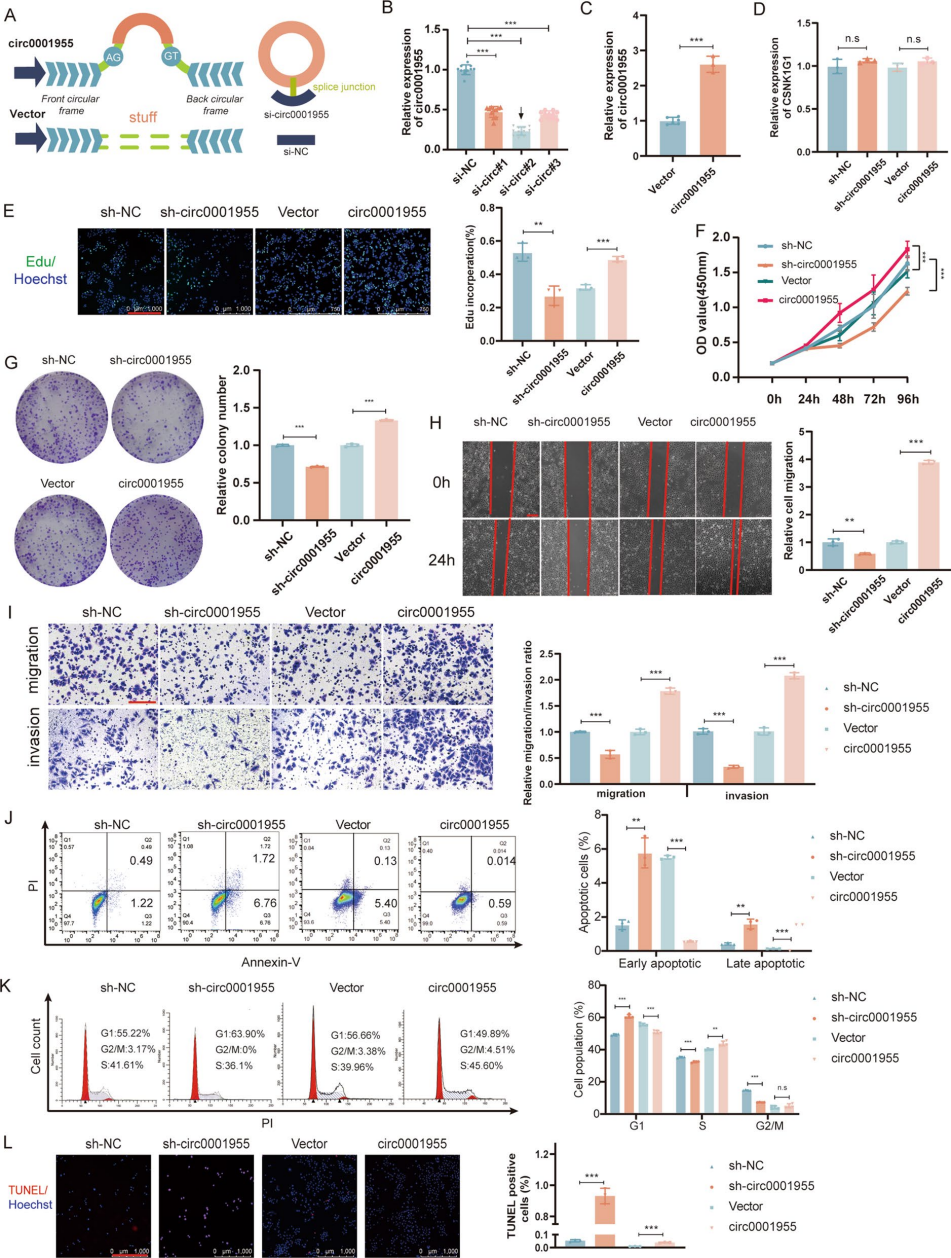
Circ0001955 promotes CSCC cell proliferation, migration and invasion. **A** Schematic illustration of circ0001955 expression vector and siRNAs. **B**, **C**, and **D** qRT-PCR analysis of circ0001955 and CSNK1G1 expression in CSCC cells transfected with si-NC, si-circ0001955, circ0001955 expression vector or vector. **E** Edu assays were conducted in cells after transfection with indicated plasmids (magnification, × 100). Scale bar, 1000 μm. **F** The growth curves of cells transfected with indicated vectors were evaluated by CCK8 assays. **G** Colony formation assays were executed to detect the proliferation of cells transfected with indicated vectors. **H** Cell migration capacities were detected by wound healing assays after transfected with indicated vectors. Scale bar, 100 μm. **I** Cell invasion abilities were determined by transwell assays after transfection. Scale bar, 200 μm. **J** Apoptosis rate was analyzed by flow cytometry after being transfected with indicated plasmids. **K** The cell cycle progression was analyzed by flow cytometry after being transfected with indicated plasmids. **L** Apoptotic cells were assessed by TUNEL after transfected with indicated plasmids. Scale bar, 1000 μm. Data were showed as mean ± SD, **P < 0.01, ***P < 0.001, *n.s* nonsignificant

To investigate the biological function of circ0001955, an overexpression vector of circ0001955 and three siRNAs targeting the junction sites of circ0001955 were constructed (Fig. 2A). The qRT‒PCR analysis demonstrated that circ0001955 was overexpressed and knocked down transfected with overexpression vector and siRNA segments respectively (Fig. 2B, C). Neither overexpression or knockdown experiments affected the expression of linear CSNK1G1 according qRT‒PCR using specific primers for linear CSNK1G1 (Fig. 2D). According to EdU assays, overexpression of circ0001955 resulted in an increase in the number of EdU-positive cells, while knockdown of circ0001955 had the opposite effect (Fig. 2E). Similarly, CCK-8 assays revealed that upregulation of circ0001955 significantly enhanced the proliferation and viability of SiHa cells, while downregulation of circ0001955 significantly inhibited cell proliferation (Fig. 2F). Additionally, colony formation assays showed that upregulation of circ0001955 enhanced the cell colony formation capabilities of SiHa cells, while downregulation of circ0001955 significantly impaired them (Fig. 2G).

To determine the effects of circ0001955 on the migration and invasion of SiHa cells, wound healing and transwell assays were conducted. The results showed that upregulation of circ0001955 significantly enhanced SiHa cell migration and invasion, while downregulation significantly suppressed these abilities (Fig. 2H, I).

### Circ0001955 induces cell cycle arrest and apoptosis

Flow cytometric analysis with Annexin V/PI double staining showed that SiHa cells transfected with sh-circ0001955 exhibited a high apoptotic rate, while overexpression of circ0001955 reduced the apoptosis rate (Fig. 2J). Furthermore, we evaluated whether circ0001955 affects the cell cycle in SiHa cells. Cell cycle analysis revealed that knockdown of circ0001955 resulted in a higher percentage of SiHa cells in G0-G1 phase and a lower percentage of cells in S phase compared with that in the control group. Upregulation of circ0001955 led to a lower percentage of cells in G0-G1 phase and a higher percentage of cells in S phase compared with that in the control group, suggesting that circ0001955 was significantly correlated with the cell cycle distribution of SiHa cells (Fig. 2K). The TUNEL assay showed that knockdown of circ0001955 significantly increased the number of TUNEL-positive cells compared with that in the control group. Conversely, upregulation of circ0001955 decreased the number of TUNEL-positive cells (Fig. 2L).

### Circ0001955 directly binds to miR-188-3p and suppresses miR-188-3p activity

**Fig. 3:**
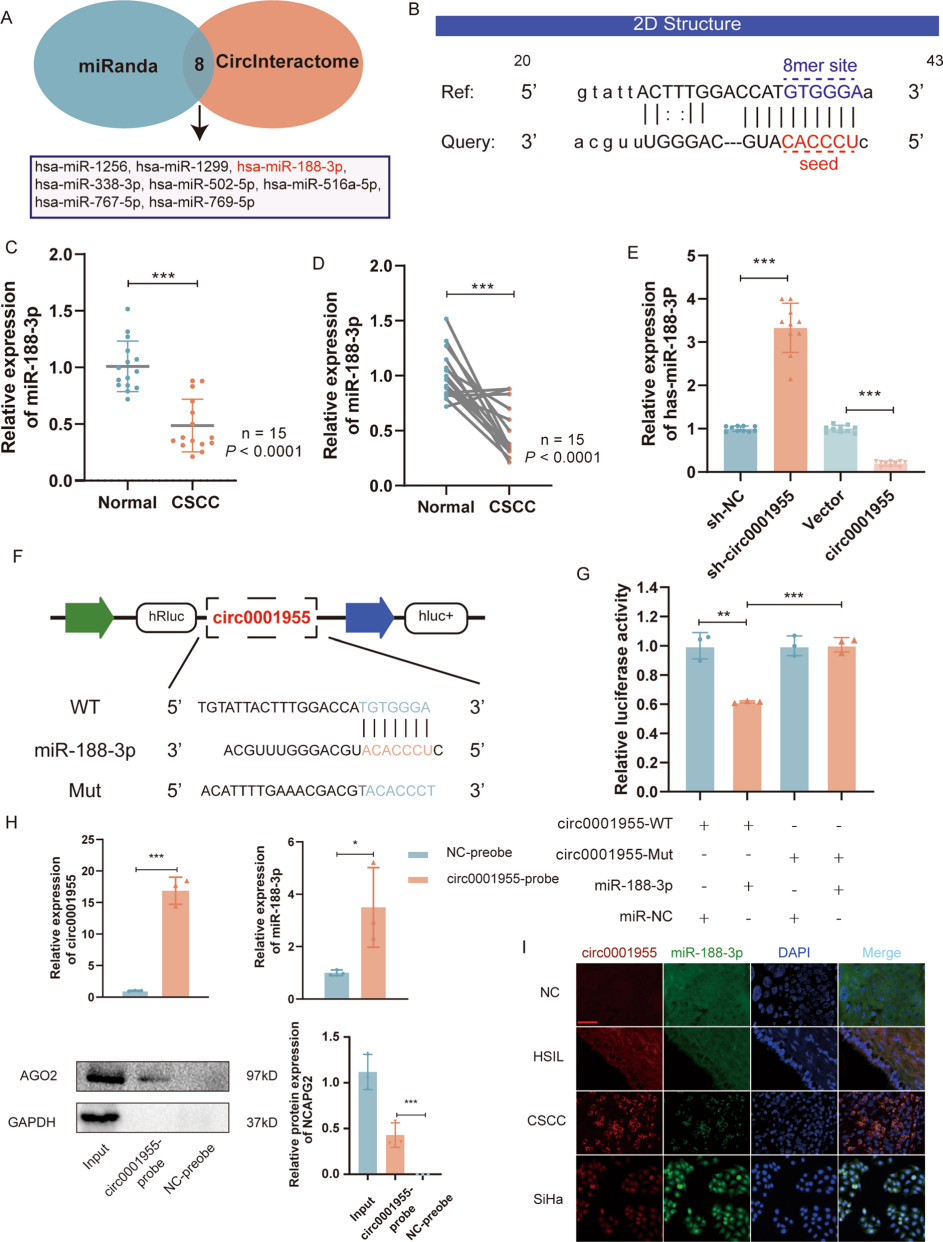
circ0001955 directly binds to miR-188-3p and suppresses miR-188-3p activity. **A** TargetScan and miRanda were utilized to predict miRNAs targeted by circ0001955. **B** The miR-188-3p binding site on circ0001955 predicted by miRanda. **C** and** D** Relative expression of miR-188-3p in CSCC tissues and adjacent non-tumor tissues (Normal) was determined by qRT-PCR (n = 15). **E** The relative expression of miR-188-3p was detected by qRT-PCR after transfection with indicated vectors. **F** Schematic illustration of circ0001955-WT and circ0001955-Mut luciferase reporter vectors. **G** The relative luciferase activities were detected in 293 T cells after transfection with circ0001955-WT or circ0001955-Mut and miR-188-3p mimics or miR-NC, respectively. **H** RAP assays showed that AGO2 and miR-188-3p could be pulled down by a biotinylated probe designed for the circ0001955 back-splicing site in SiHa cells. **I** FISH was performed to observe the cellular location of circ0001955 (red) and miR-188-3p (green) in tissues. Scale bar, 100 μm. Data were showed as mean ± SD, *P < 0.05, **P < 0.01, ***P < 0.001, n.s, nonsignificant

To explore the underlying molecular mechanism of circ0001955, first, bioinformatic analysis was implemented to predict the possible binding sites of circ0001955 and miR-188-3p with the miRanda database (http://www.microrna. org) [18] and TargetScan database (http://www.targetscan.org) [19] (Fig. 3A). Combination of sequence matching, functional analyses, and correlations with cancer led to the identification of 8 miRNAs, in which miR-188-3p has a high binding score with circ0001955 according to the CircInteractome database (Fig. 3B) and its reported role in cancer, which makes it a promising candidate for further investigation. In contrast, miR-644 and miR-516a-5p, although they showed high binding scores with circ0001955, were inconsistent with the prognosis of cervical cancer patients as expected (Additional file 5). The levels of miR-188-3p were then examined in 15 pairs of CSCC tissues and adjacent noncancerous tissues, and the results indicated that miR-188-3p expression was markedly lower in CSCC tissues than in adjacent nontumor tissues (Fig. 3C, D). Pearson correlation analysis revealed that the expression of circ0001955 was reversely correlated with the level of miR-188-3p in CSCC tissues (Additional file 3: Fig. S2A). Based on TCGA data, Kaplan‒Meier survival analysis showed that miR-188-3p expression correlated strongly with patient overall survival (Additional file 3: Fig. S2B). Therefore, we supposed that circ0001955 might serve as a ceRNA for miR-188-3p. Next, qRT‒PCR experiments were conducted to examine the impact of circ0001955 on the expression of miR-188-3p. The results showed that knockdown of circ0001955 resulted in upregulation of miR-188-3p and that overexpression of circ0001955 led to downregulation of miR-188-3p in SiHa cells (Fig. 3E).

Subsequently, dual-luciferase reporter assays were applied to confirm the binding between circ0001955 and miR-188-3p. The wild-type (WT) and mutant dual-luciferase reporter plasmids of circ0001955 were constructed (Fig. 3F). The data indicated that miR-188-3p mimics obviously reduced the luciferase activity of the circ0001955-WT luciferase reporter but not that of mutants (Fig. 3G), which suggests that circ0001955 might directly combine with miR-188-3p. To further confirm the binding of circ0001955 with miR-188-3p, a biotinylated circ0001955 probe was used to conduct RAP experiments, which revealed that circ0001955 could bind to the AGO2 protein and that miR-188-3p was significantly enriched in RNAs retrieved from the circ0001955 complex (Fig. 3H). Additionally, RNA FISH revealed that circ0001955 and miR-188-3p colocalized in the cytoplasm (Fig. 3I). Together, these data suggest that circ0001955 could directly combine with miR-188-3p and act as a competitive inhibitor of miR-188-3p.

### NCAPG2 is directly targeted by miR-188-3p and indirectly regulated by circ0001955

**Fig. 4:**
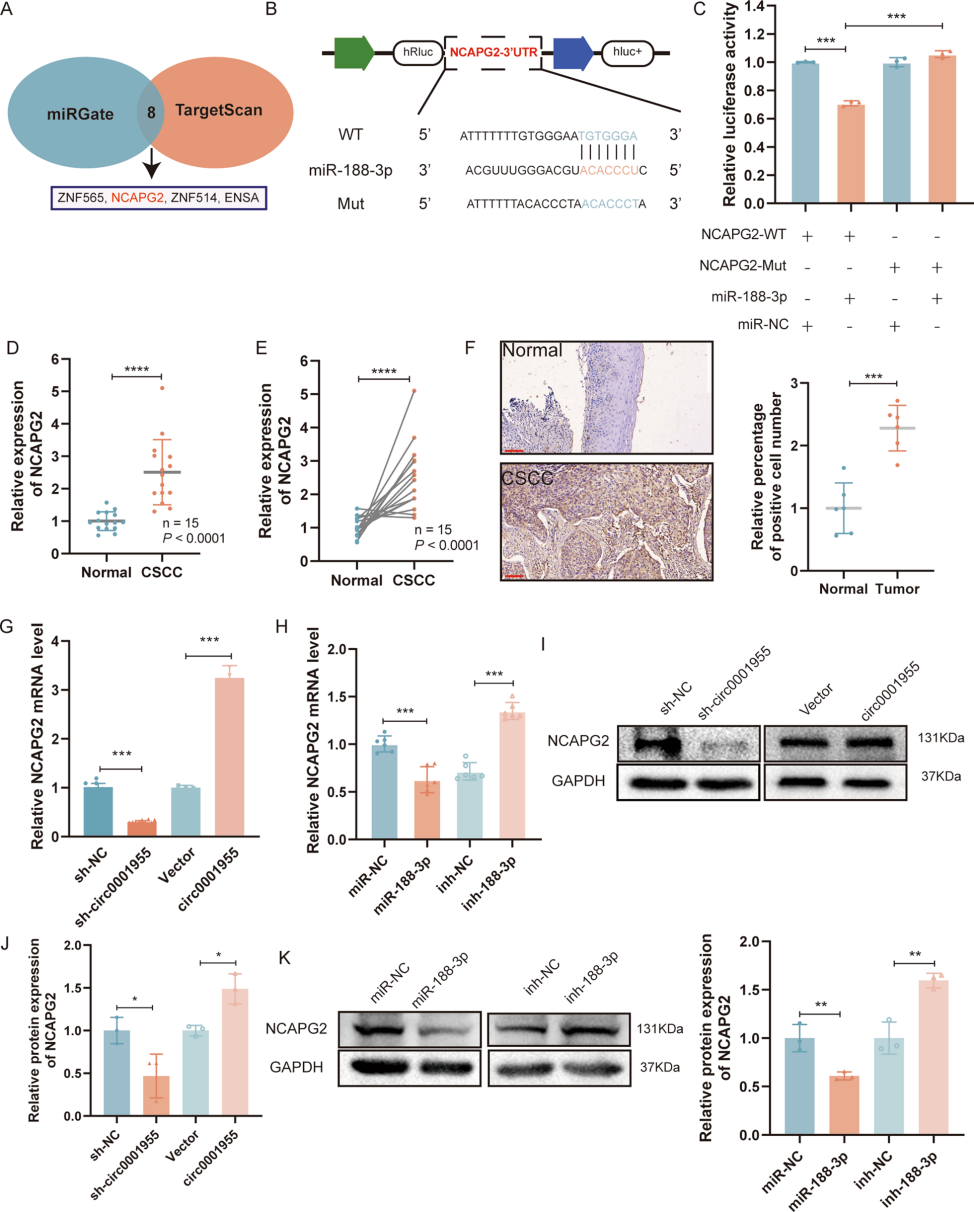
NCAPG2 is directly targeted by miR-188-3p and indirectly regulated by circ0001955. **A** miRGate and TargetScan were utilized to predict mRNAs targeted by miR-188-3p. **B** Schematic illustration of NCAPG2-WT and NCAPG2-Mut luciferase reporter vectors. **C** The relative luciferase activities were detected in 293 T cells after transfection with NCAPG2-WT or NCAPG2-Mut and miR-188-3p mimics or miR-NC, respectively. **D** Relative expression of NCAPG2 in CSCC tissues and adjacent non-tumor tissues (Normal) were detected by qRT-PCR (n = 15). **F** Representative images of NCAPG2 IHC staining in CSCC tissues and adjacent non-tumor tissues (Normal). Scale bar, 100 μm. **G** and **H** The relative expression of NCAPG2 mRNA was detected by qRT-PCR after transfection with indicated vectors. **I**, **J** and **K** NCAPG2 protein expression in SiHa cells transfected with indicated vectors. Data were showed as mean ± SD, *P < 0.05, **P < 0.01, ***P < 0.001,****P <0.0001

According to TargetScan (http://www.targetscan.org/vert_72/) and miRGate (http://mirgate.bioinfo.cnio.es/API/api.html), NCAPG2 is a cell cycle-related gene related to circ0001955. In addition, circ0001955 and miR-188-3p share an mRNA response element (MRE) (Fig. 4A). A dual-luciferase reporter assay was conducted to validate this prediction and revealed that miR-188-3p mimics significantly inhibited the activity of the luciferase reporter vector carrying the NCAPG2 3’UTR-WT sequence compared with that of the control vector (Fig. 4B, C). To determine whether NCAPG2 is co-overexpressed with circ0001955, the levels of NCAPG2 were examined in the 15 pairs of CSCC tissues and para-cancerous tissues by qRT‒PCR (Fig. 4D, E) and IHC (Fig. 4F). Pearson correlation analysis indicated that the expression levels of circ0001955 were positively associated with those of NCAPG2 (Additional file 3: Fig. S2C). Then, analysis of RNA-seq data for 306 CSCC tissues and 3 nontumor tissues obtained from The Cancer Genome Atlas (TCGA) further confirmed that NCAPG2 was upregulated in CSCC tissues compared with normal tissues (Additional file 3: Fig. S2D). Further Kaplan‒Meier survival curve analysis based on TCGA data showed that a higher level of NCAPG2 was correlated with disease-free survival (DFS) (HR = 4.09, P = 0.038) (Additional file 3: Fig. S2E). These results confirmed the robustness of our microarray data and suggest that circ0001955 and NCAPG2 might participate in the tumorigenesis and development of CSCC. qRT‒PCR demonstrated that silencing circ0001955 significantly lowered the expression level of NCAPG2 and that overexpression of circ0001955 dramatically increased NCAPG2 expression levels in SiHa cells (Fig. 4G). Moreover, miR-188-3p mimics decreased the expression of NCAPG2, while miR-188-3p inhibitors significantly increased the level of NCAPG2 in SiHa cells (Fig. 4H). As illustrated in Fig. 4I–K, NCAPG2 protein levels in SiHa cells were accordingly altered.

### Circ0001955 promotes SiHa cell proliferation and invasion and activates AKT-mTOR pathway through the circ0001955/miR-188-3p/NCAPG2 axis

**Fig. 5:**
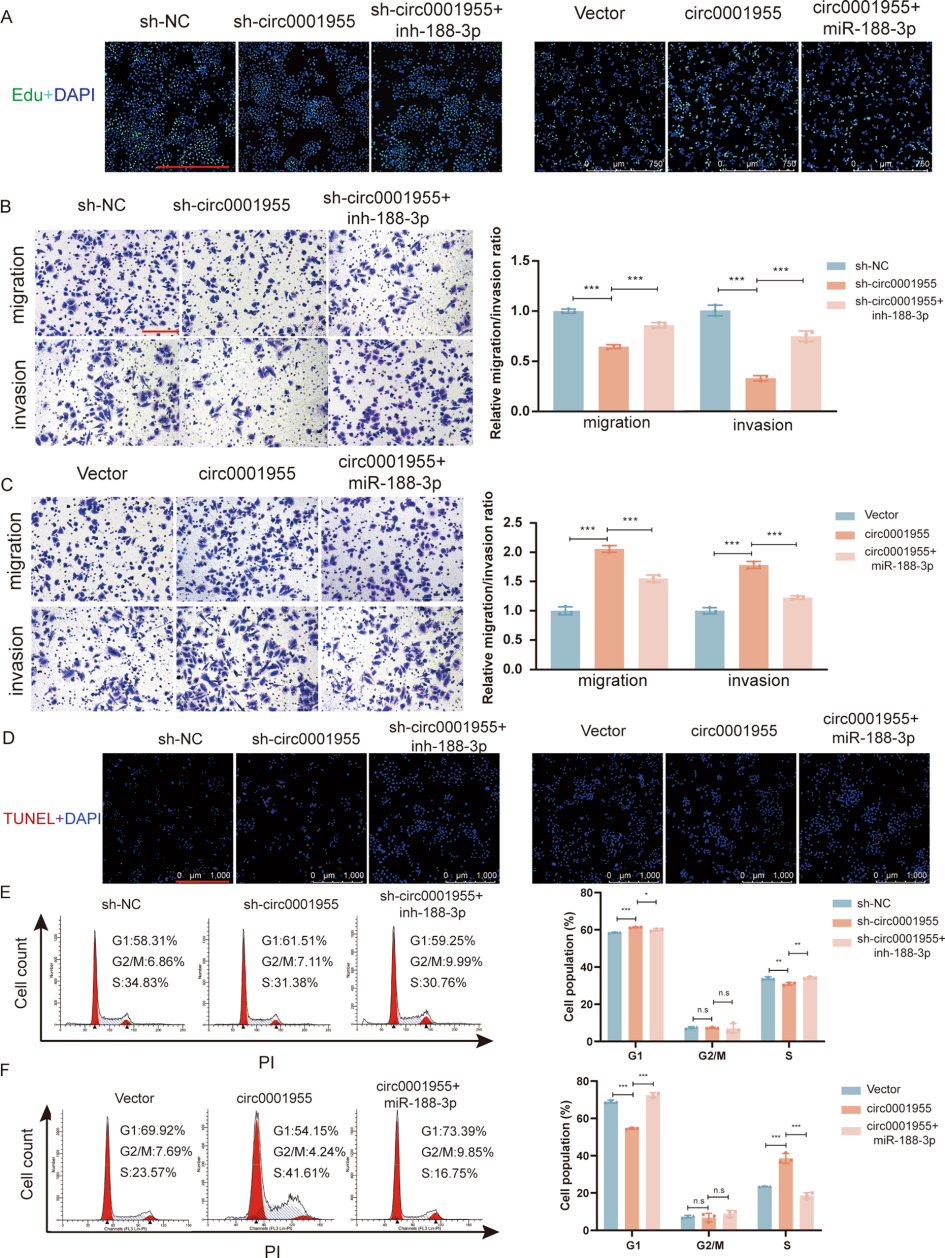
Circ0001955 promotes SiHa cell proliferation and invasion and activates AKT-mTOR pathway through the circ0001955/miR-188-3p/NCAPG2 axis **A** The proliferative abilities of SiHa cells were detected by Edu assays in the indicated cells. Original magnification, × 100. Scale bar, 750 μm. **B** and **C** Transwell assays were performed to investigate the migration and invasion abilities of SiHa cells in the indicated groups. Original magnification, × 100. Scale bar, 200 μm. **D** Apoptotic cells were assessed by TUNEL after transfected with indicated plasmids. Scale bar, 1000 μm. **E** and **F** The cell cycle progression was analyzed by flow cytometry after being transfected with indicated plasmids. *P < 0.05, **P < 0.01, ***P < 0.001.

Rescue experiments using miR-188-3p inhibitors and mimics were designed to investigate whether circ0001955 serves its biological function through the circ0001955/miR-188-3p/NCAPG2 axis. As shown by EdU and transwell assays, miR-188-3p inhibitors restored the proliferation, migration and invasion suppressing effect of circ0001955 knockdown in SiHa cells, whereas the overexpression of circ0001955 rescued miR-188-3p mimic-mediated promotion of these cellular phenotypes (Fig. 5A–C, Additional file 3: Fig. S2F, G). Moreover, cell cycle arrest was obviously increased and apoptosis was significantly augmented by circ0001955 depletion; miR-188-3p inhibitors restored cell viability, reflected by a low level of apoptosis and reduced cell cycle arrest; and the presence of circ0001955 restored cell cycle progression and reduced apoptosis (Fig. 5D–F, Additional file 3: Fig. S2H).

**Fig. 6:**
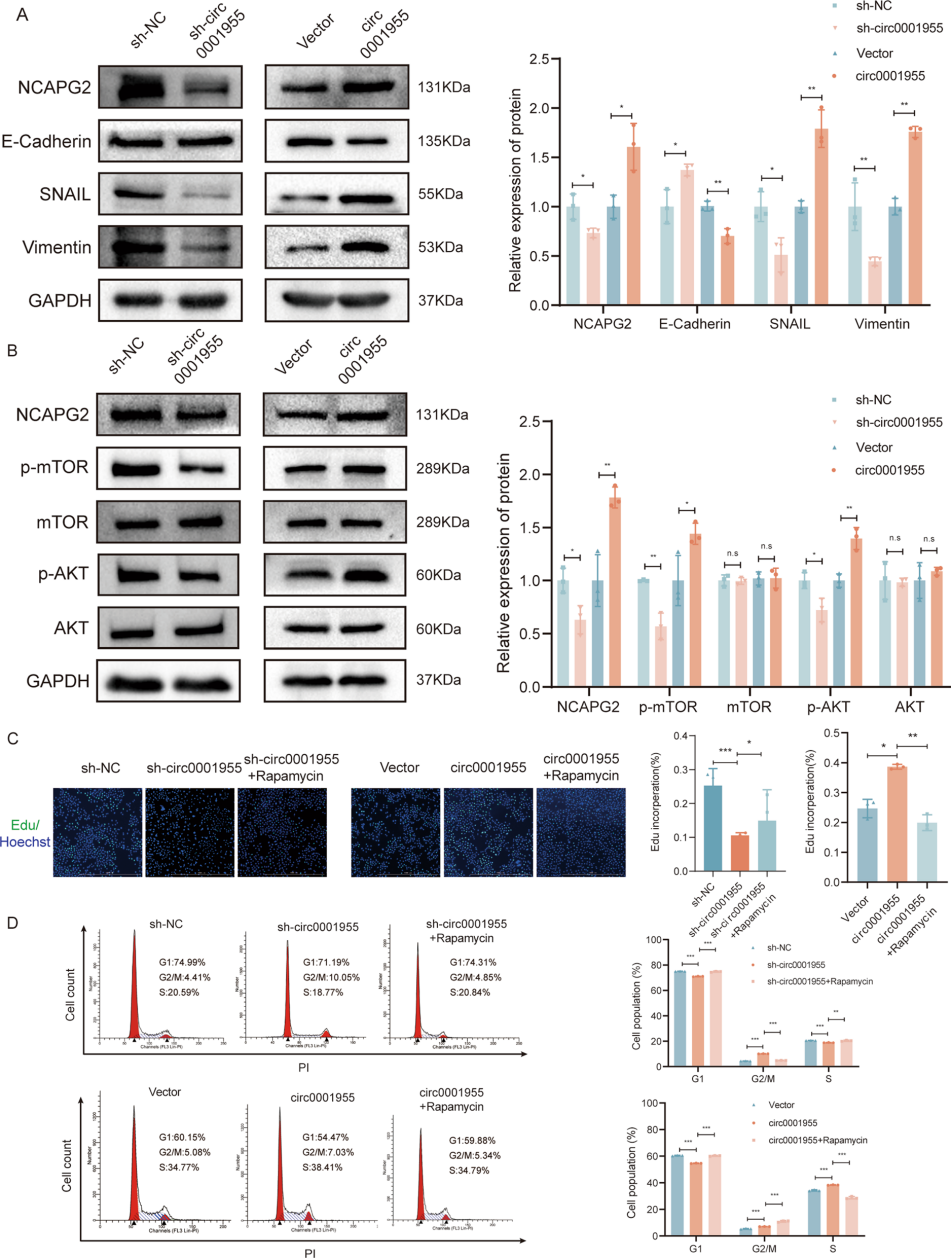
Circ0001955 is associated with CSCC EMT and responds to mTOR inhibitor treatment **A** Western blot analysis of the protein levels of NCAPG2 and various EMT markers Snail, E-cadherin, and Vimentin in the indicated cells. **B** Western blot analysis of the protein levels of AKT/mTOR pathway and NCAPG2 in the indicated cells. **C** The proliferative abilities of SiHa cells were detected by Edu assays in the indicated cells. Original magnification, × 100. Scale bar, 1000 μm.** D** The cell cycle progression was analyzed by flow cytometry after being transfected in indicated cells. Each experiment was performed at least three times independently. Rapamycin: 500 nM. *P < 0.05, **P < 0.01, ***P < 0.001, *n.s* nonsignificant

NCAPG2, a chromatin remodeling protein, has been reported to be highly expressed in numerous cancers [20]. NCAPG2 has been shown to promote proliferation, migration, invasion, and epithelial–mesenchymal transition (EMT) in various cancers, such as liver cancer and non-small cell lung cancer [21, 22]. We observed that the depletion of NCAPG2 significantly increased the levels of E-cadherin but decreased the levels of Snail and vimentin in SiHa cells while suppressing the activation of the AKT/mTOR pathway in CSCC cells (Fig. 6A, B). Moreover, the overexpression of circ0001955 led to increased levels of NCAPG2 and enhanced EMT, and activated AKT/mTOR pathway in SiHa cells (Fig. 6A, B). mTOR is a critical activator of the PI3K/AKT signaling pathway. Targeting mTOR has been demonstrated as an effective strategy to inhibit tumor progression. Similarly, our study found that mTOR inhibition using rapamycin attenuated cell proliferation in SiHa cells (Additional file 4: Fig. S3A–B), which corresponded with reduced levels of phosphorylated mTOR protein (Additional file 4: Fig. S3C). Given that our results indicated circular RNA circ0001955 promoted mTOR expression in CSCC, we further investigated whether the oncogenic effects of circ0001955 could be mitigated by suppressing mTOR. In vitro experiments suggested that rapamycin could reverse the impaired cell proliferation (Fig. 6C) and cell cycle arrest (Fig. 6D) induced by circ0001955 knockdown, vice versa. These findings demonstrate that circ0001955 promotes CSCC progression through an mTOR-dependent pathway, which can be abolished by treatments with AKT/mTOR signaling pathway inhibitors.

### Circ0001955 accelerates the growth of xenograft tumors in vivo

**Fig. 7:**
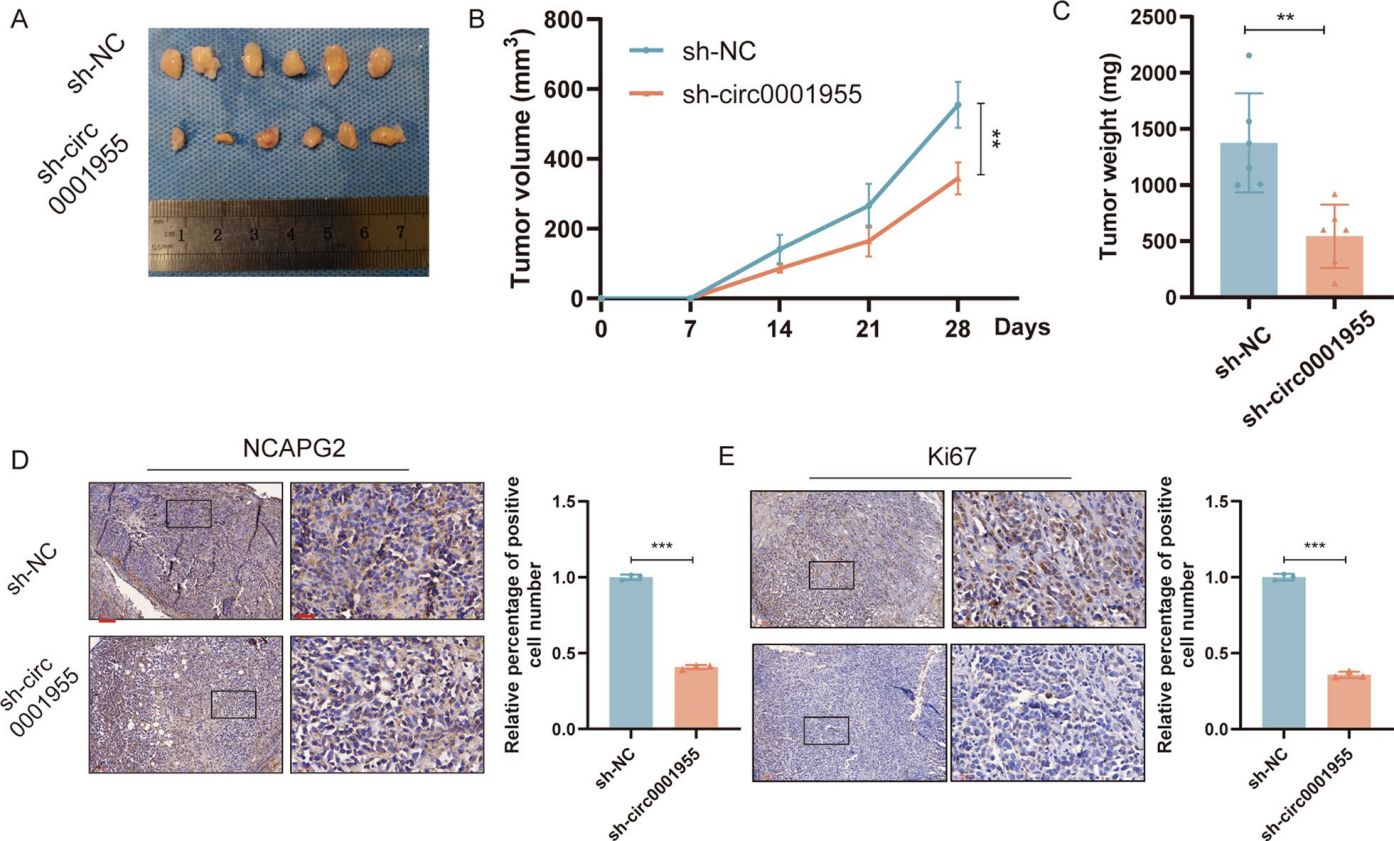
Circ0001955 accelerates the growth of xenograft tumors in vivo **A** Representative image of dissected tumors from nude mice transplanted with sh-circ0001955 or sh-NC. **B** Subcutaneous tumor growth curves of mice in different treatment groups. **C** The mean weight of tumors upon sacrifice in the experimental groups. **D** Representative images of NCAPG2 IHC staining different treatment groups. Scale bar, 100 μm and 30 μm. **E** Representative images of Ki67 IHC staining different treatment groups. Scale bar, 100 μm and 30 μm. Each experiment was performed at least three times independently. **P < 0.01

To evaluate whether circ0001955 affected in vivo tumor growth, a human CSCC xenograft model was established. SiHa cells with stable knockdown of circ0001955 and control cells were subcutaneously injected into female nude mice. The results showed that the tumors derived from knockdown group were slower growth, smaller and weighed less compared to vector group (Fig. 7A–C).

We further explored the impact of circ0001955 on the expression of the target gene NCAPG2 in vivo. IHC analysis revealed that silencing circ0001955 could decrease the expression of NCAPG2 in tumor tissues (Fig. 7D). Furthermore, the proportion of proliferating cells (Ki67 +) were significantly decreased after circ0001955 was knocked down (Fig. 7E). These results were consistent with those of in vitro assays, suggesting that circ0001955 could promote tumorigenesis of CSCC by activating NCAPG2.

## Discussion

With the development of RNA sequencing and bioinformatics technologies, a host of circRNAs have been implicated in multiple malignancies. Despite this, studies of circRNA functions are still in their infancy, and the mechanisms by which circRNAs influence the progression of CSCC have yet to be determined. In this study, we uncovered and investigated the significance of circ0001955 in CSCC tumor growth and metastasis. We found that the gain of circ0001955 expression was closely associated with the progression of CSCC. Moreover, we found that circ0001955 promoted tumor proliferation and invasion in CSCC by modulating the expression of NCAPG2. At the mechanistic level, we identified that circ0001955 restored the expression of NCAPG2 by competitive binding miR-188-3p and actives AKT/mTOR pathway. This study provides new insights into the role of circRNAs in CSCC progression and highlights the potential for therapeutic application of circ0001955 for the diagnosis and treatment of CSCC.

CircRNAs exhibit dynamic cell/tissue-specific expression and play critical roles in multiple cellular processes, functioning as miRNA competitive inhibitor, partners of RNA-binding proteins or encode polypeptides [23–29]. Despite this, it is now widely accepted that circRNAs can effectively competitive inhibitor miRNAs due to the presence of high-affinity miRNA-binding sites [30–32]. Previous literature has indicated that circRNAs might serve as ceRNAs to modulate the development and progression of CSCC [33]. Based on bioinformatics analyses, we found that circ0001955 contained the MRE of miR-188-3p. FISH assays showed that circ0001955 and miR-188-3p were colocalized in the cytoplasm of SiHa cells. Therefore, we concluded that circ0001955 competitive binding miR-188-3p might play an oncogenic role in CSCC. Furthermore, dual-luciferase reporter, nucleocytoplasmic separation and RAP confirmed that circ0001955 could directly bind miR-188-3p. In addition, miR-188-3p was significantly downregulated in CSCC tissues and positively correlated with patient overall survival based on TCGA data. miR-188-3p is significantly downregulated in breast cancer [34], colorectal cancer [35], hepatocellular carcinoma [36] and pancreatic cancer [37] and negatively correlated with the degree of malignancy. Moreover, miR-188-3p suppresses proliferation, invasion, and metastasis in several cancers by targeting multiple genes [21]. However, the function of miR-188-3p in CSCC remains unexplored. Our findings showed that circ0001955 serves as an oncogene in CSCC by competitive binding miR-188-3p and indicated the vital role of the interaction between circ0001955 and miR-188-3p in tumorigenesis and development of CSCC.

It has been hypothesized that circRNAs can act as ceRNAs to modulate the expression of miRNA target genes [38]. It was found that NCAPG2, a mitosis and cell cycle regulator [39], is co-overexpressed in CSCC. Interestingly, the cell cycle was the pathway most significantly enriched in both Gene Ontology (GO) and Kyoto Encyclopedia of Genes and Genomes (KEGG) pathway analyses, supporting the notion that the cell cycle is closely associated with CSCC tumorigenesis and progression. Furthermore, a bioinformatics analysis using miRGate and TargetScan indicated that NCAPG2 is a potential target of miR-188-3p. Then, using a dual-luciferase reporter assay, miR-188-3p was confirmed to target the 3′-untranslated region of NCAPG2. Additionally, miR-188-3p upregulation led to the downregulation of NCAPG2 mRNA and protein, whereas miR-188-3p downregulation produced the opposite effect. We found that downregulation of circ0001955 resulted in G1/S phase cell cycle arrest. NCAPG2 contributes to tumor proliferation and is associated with poor prognosis among lung adenocarcinomas [22]. Mechanistically, NCAPG2 plays a crucial role in facilitating the kinetochore localization of PLK1 during mitosis, which in turn is essential for the proper alignment and segregation of chromosomes [39]. Moreover, PLK1 is responsible for orchestrating the degradation of Claspin required for DNA damage checkpoint recovery, and its activation is tightly regulated by the phosphorylation status of Aurora A. In the context of HPV-induced cervical cancer, it has been observed that cells expressing HPV-16 E7 exhibit elevated levels of both Aurora A and PLK1, leading to the possibility of dysregulated regulation of these kinases and cell cycle checkpoints. Consequently, it is conceivable that dysregulation of NCAPG2 and PLK1 localization and regulation, possibly mediated by HPV-16 E7, may contribute to the pathogenesis and progression of cervical cancer [40]. However, it remains to be determined how NCAPG2 plays a role in CSCC progression. In accordance with previous studies, we observed that NCAPG2 overexpression in CSCC tissues was associated with shorter DFS time in patients. We next examined the crosstalk between circ0001955 and NCAPG2, and we found that knockdown of circ0001955 decreased the NCAPG2 mRNA and protein levels. Moreover, these effects could be partially reversed by miR-188-3p inhibitors, which may support our hypothesis that circ0001955 acts as a ceRNA to promote NCAPG2-mediated proliferation and metastasis by acting as a decoy of miR-188-3p in CSCC.

Another significant finding in our study was that circ0001955 could activate the AKT/mTOR pathway. In CSCC, the AKT/mTOR pathway is often abnormally activated, especially in metastatic CSCC [41]. AKT/mTOR signaling hyperactivation promotes the proliferation, metastasis, and recurrence of AKT/mTOR. While several studies have revealed sophisticated regulatory networks involving the AKT/mTOR pathway, it is unclear whether circRNAs play a role in CSCC AKT/mTOR activation. As we found, circ0001955 promoted the phosphorylation of AKT and mTOR, important components of the AKT/mTOR pathway, which resulted in the downstream EMT process. Furthermore, rapamycin, an mTOR inhibitor, inhibited the progression of CSCC induced by circ0001955. As a result of these findings, it can be concluded that circRNA functions as a key regulator of the AKT/mTOR pathway and can be used as a potential target for intervention in CSCC.

In summary, we found that circ0001955 was overexpressed in CSCC. We also demonstrated that circ0001955 promoted the proliferation and metastasis of CSCC through the miR-188-3p/NCAPG2 axis-mediated activation of the AKT/mTOR pathway. We not only provide insight into the role of circRNAs in the development and progression of CSCC, but also review circ0001955 as a potential prognostic biomarker and promising therapeutic target for CSCC.

## Conclusions

In summary, we demonstrate that increased expression of circ0001955 is a frequent occurrence during the progression of CSCC. We first demonstrated that circ0001955 might competitively bind miR-188-3p to modulate NCAPG2 expression and activate the AKT-mTOR signaling pathway, leading to tumorigenesis and the development of CSCC. We suggest that circ0001955 may be a useful prognostic marker for CSCC and a potential diagnostic marker and treatment target in the future. The regulatory network involving the circ0001955/miR-188-3p/NCAPG2 axis might provide a better understanding of the potential mechanism of the pathogenesis and progression of CSCC.

## Supplementary Information

**Additional file 1: Table S1** Sequences of primers and siRNA target site used in the study.

**Additional file 2:** **Figure S1 ** Screening upregulated circular RNAs in CSCC tissues.

**Additional file 3:** **Figure S2** Expression relationship and function of circ0001955 with miR-188-3p and NCAPG2.

**Additional file 4:** **Figure S3** Rapamycin can inhibit the proliferation of CSCC and the activation of AKT/mTOR pathway.

**Additional file 5:** TargetScan database predicts potential target miRNAs of circ0001955 and Kaplan-Meier survival curve of overall survival based on TCGA database with CSCC according to the miR-644a and miR-516a expression.

## Data Availability

Not applicable.

## Abbreviations

circRNAs: Circular RNAs
CSCC: Cervical squamous cell carcinoma
IHC: Immunohistochemistry
qRT-PCR: Real-time quantitative polymerase chain reaction
HE: Hematoxylin and eosin
HSIL: High-grade squamous intraepithelial lesion
LSIL: Low-grade squamous intraepithelial lesion
siRNAs: Small interfering RNAs
ceRNAs: Competitive endogenous RNAs
EMT: Epithelial mesenchymal transformation
AKT: Protein kinase B
mTOR: Mechanistic target of rapamycin
FISH: Fluorescence in situ hybridization
RAP: RNA antisense purification
AGO2: Argonaute 2
PLK1: Polo-like kinase 1
