# Supplementary material for: Circular RNA circ0001955 promotes cervical cancer tumorigenesis and metastasis via the miR-188-3p/NCAPG2 axis
Source: J Transl Med. 2023 May 29;21:356. doi: 10.1186/s12967-023-04194-4 (PMC10226249; doi:10.1186/s12967-023-04194-4)
Supplement: Supplementary file 2 — Additional file 2: Figure S1 Screening upregulated circular RNAs in CSCC tissues. [file 12967_2023_4194_MOESM2_ESM.docx]

**
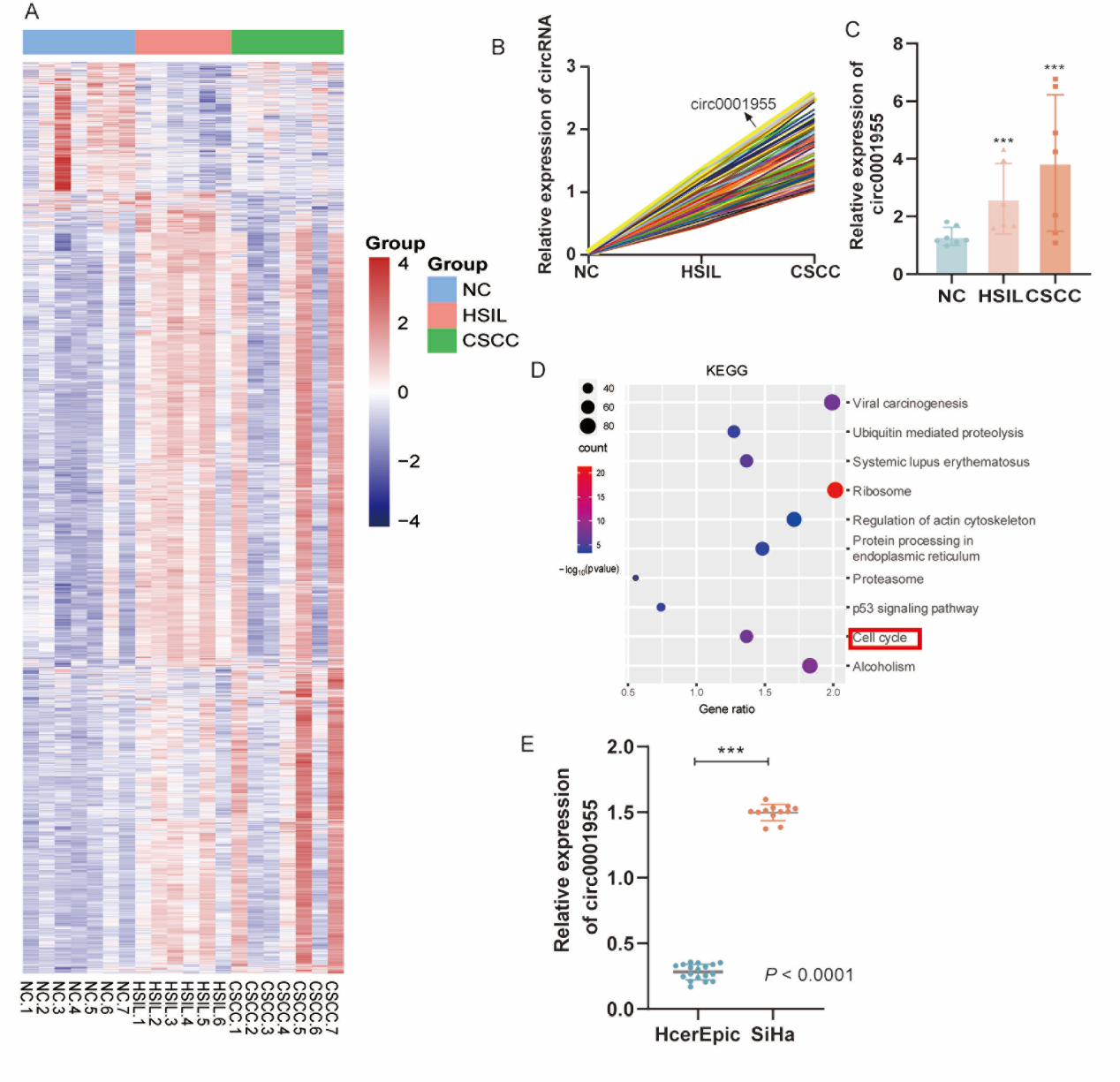
Figure S1 A** Heatmap of differentially expressed circRNAs in the three comparison groups. **B and C** Expression of circ0001955 is gradually upregulated during the process of CSCC. **D** KEGG pathway analysis of differentially expressed mRNAs. **E** Relative expression of circ0001955 in cell lines was determined by qRT-PCR. ***P<0.001.
