# Supplementary figures and images for: Circular RNA circ0001955 promotes cervical cancer tumorigenesis and metastasis via the miR-188-3p/NCAPG2 axis
Source: J Transl Med. 2023 May 29;21:356. doi: 10.1186/s12967-023-04194-4 (PMC10226249; doi:10.1186/s12967-023-04194-4)

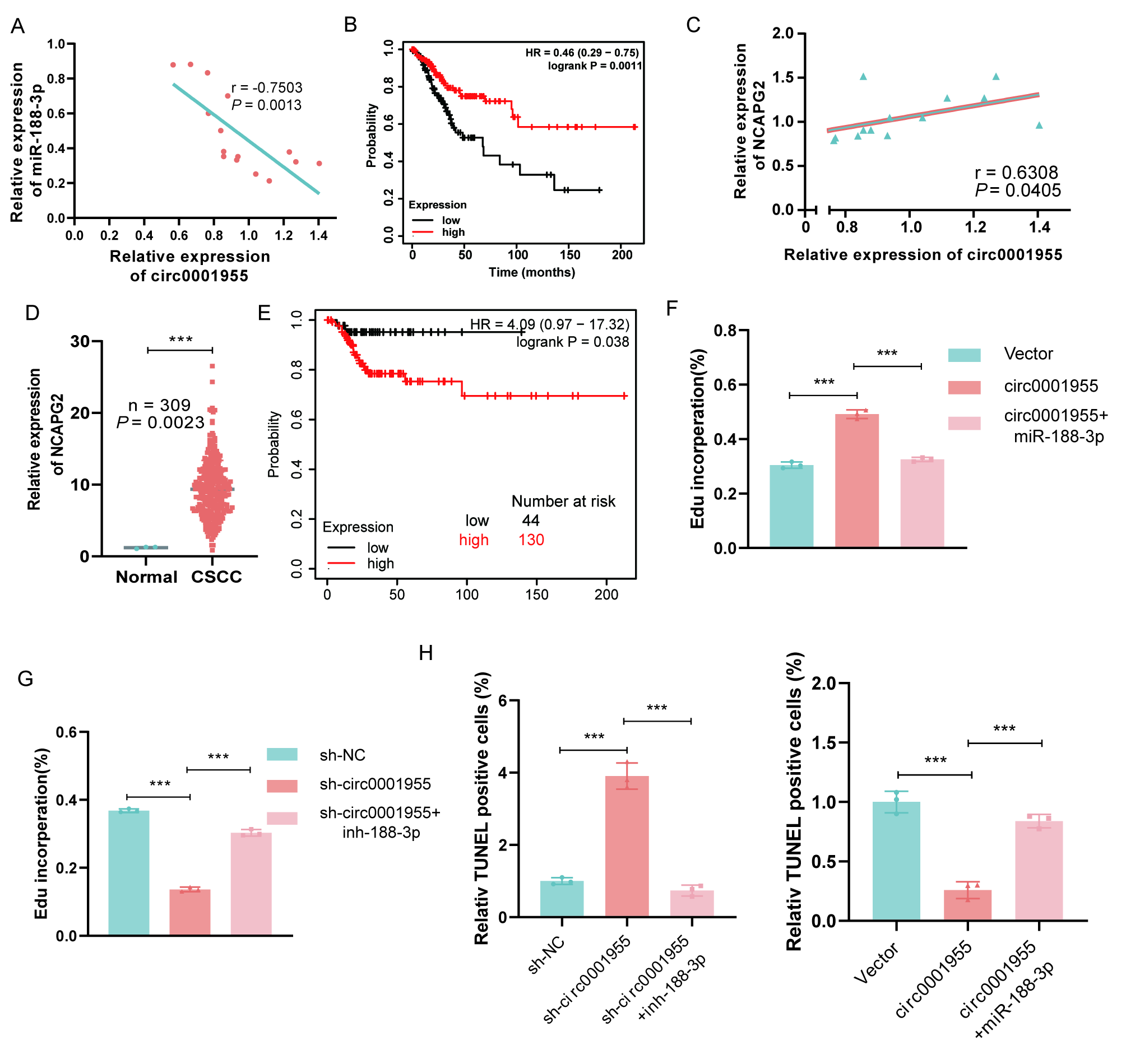

Supplement: Supplementary file 3 — Additional file 3: Figure S2 Expression relationship and function of circ0001955 with miR-188-3p and NCAPG2. [file 12967_2023_4194_MOESM3_ESM.tif]

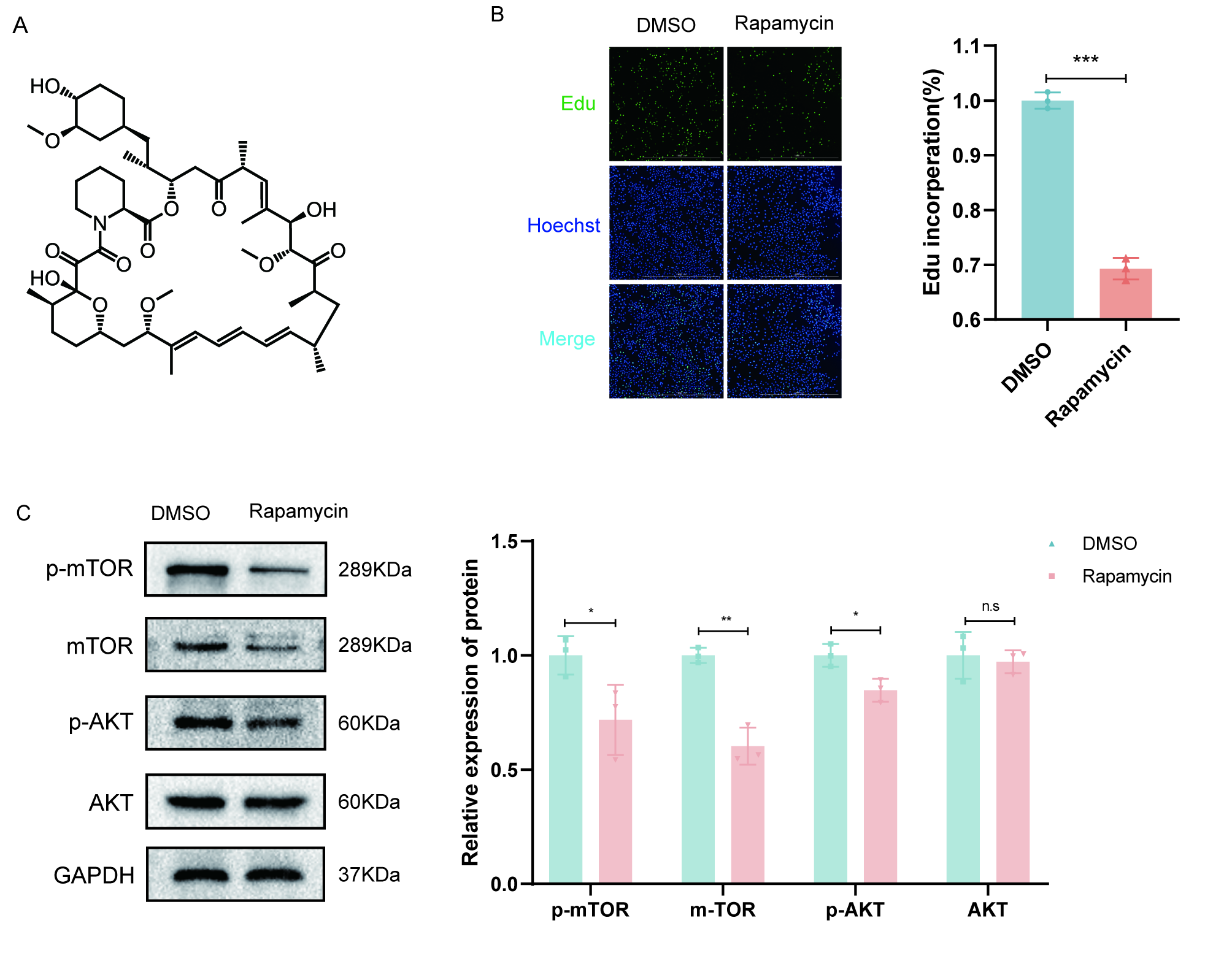

Supplement: Supplementary file 4 — Additional file 4: Figure S3 Rapamycin can inhibit the proliferation of CSCC and the activation of AKT/mTOR pathway. [file 12967_2023_4194_MOESM4_ESM.tif]

## Slide 1
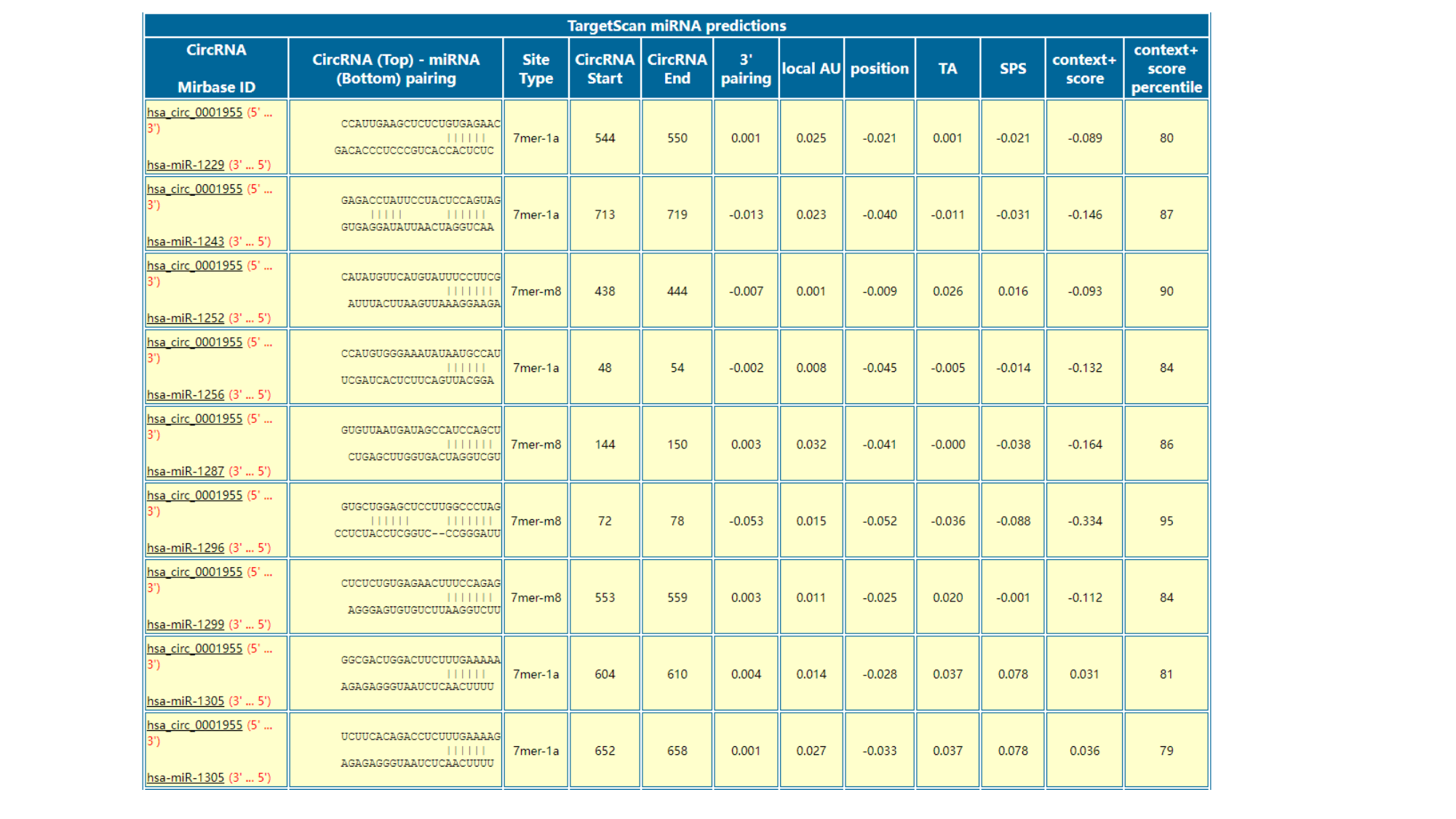

#

## Slide 2
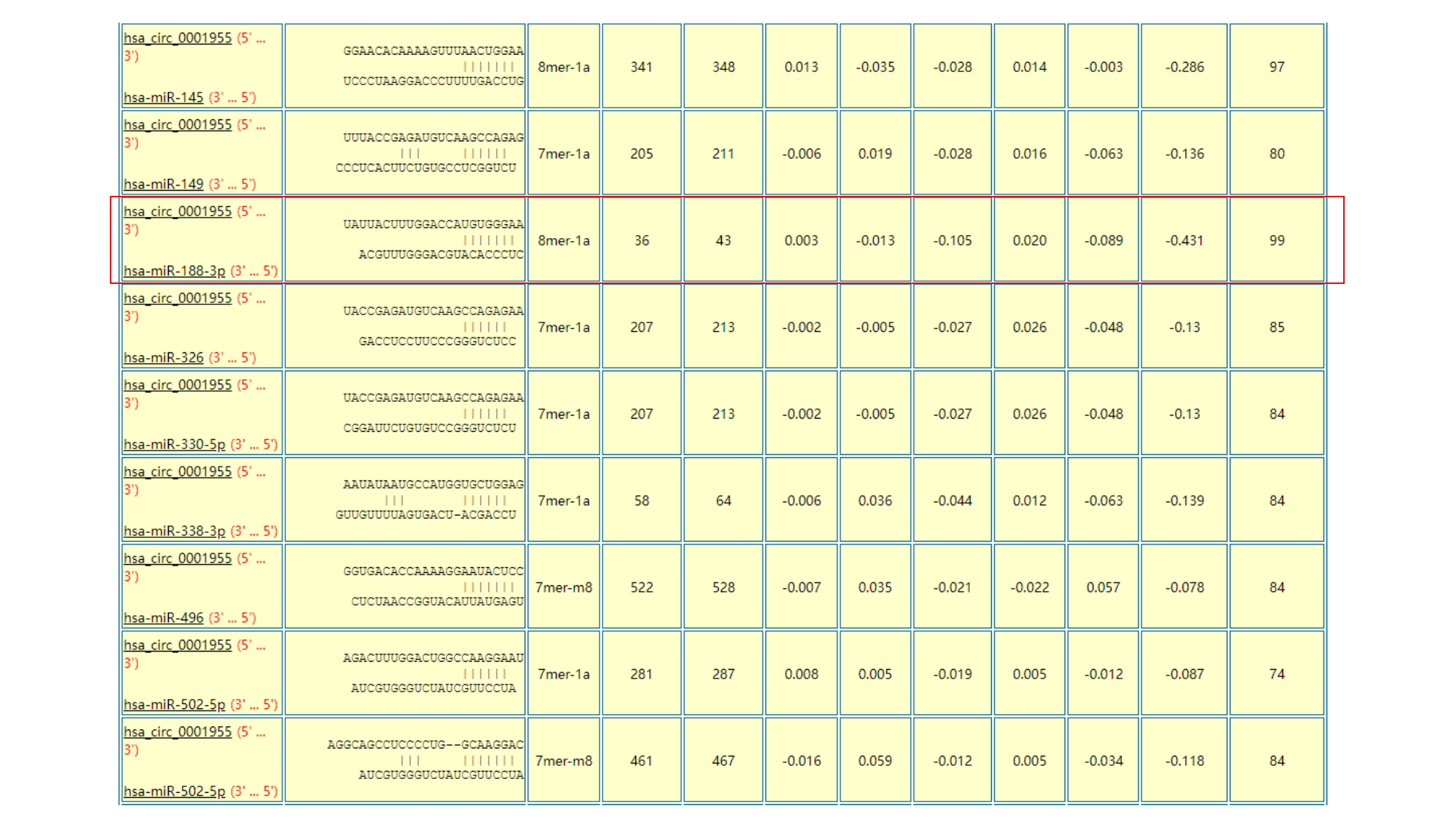

#

## Slide 3
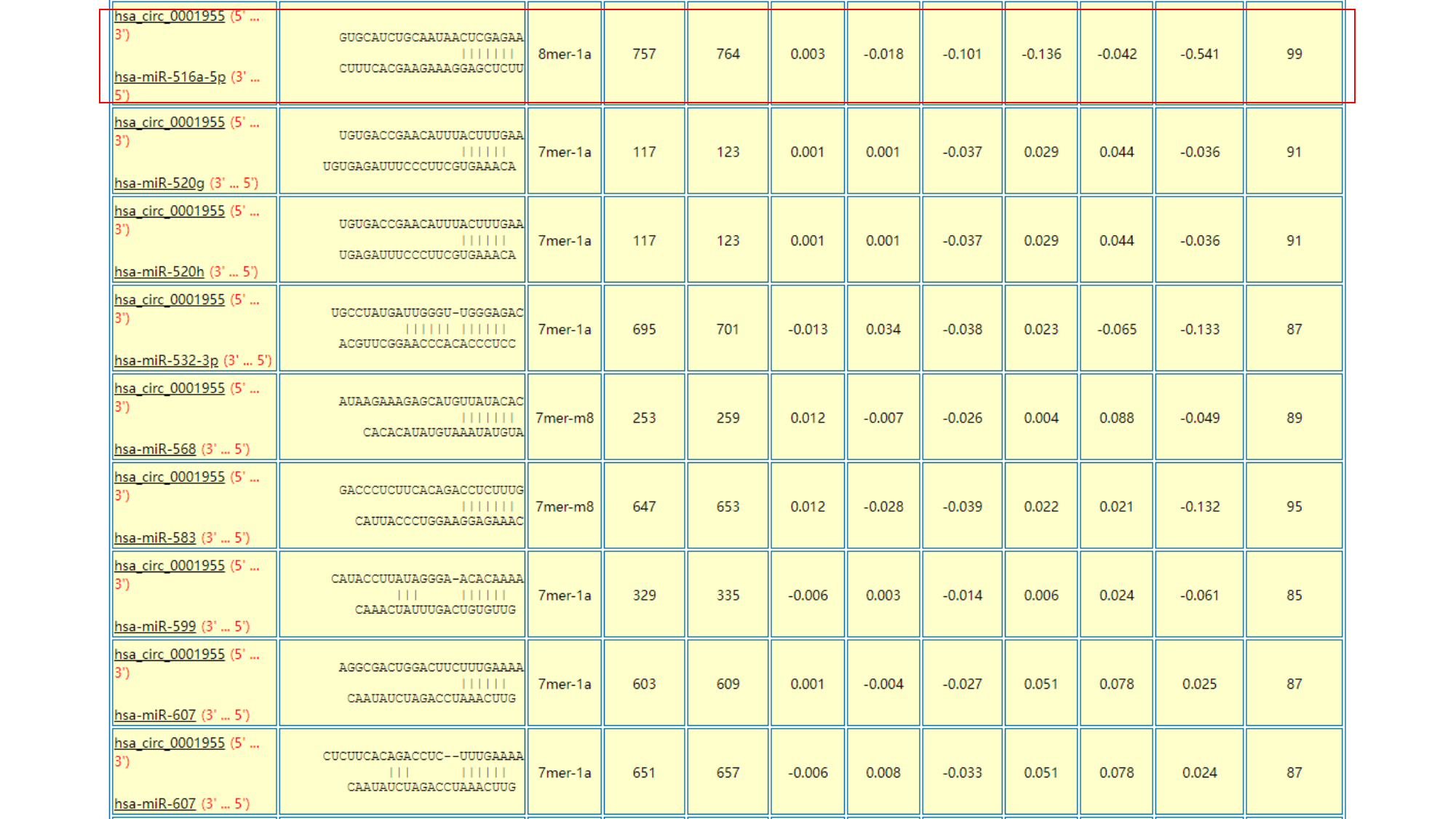

#

## Slide 4
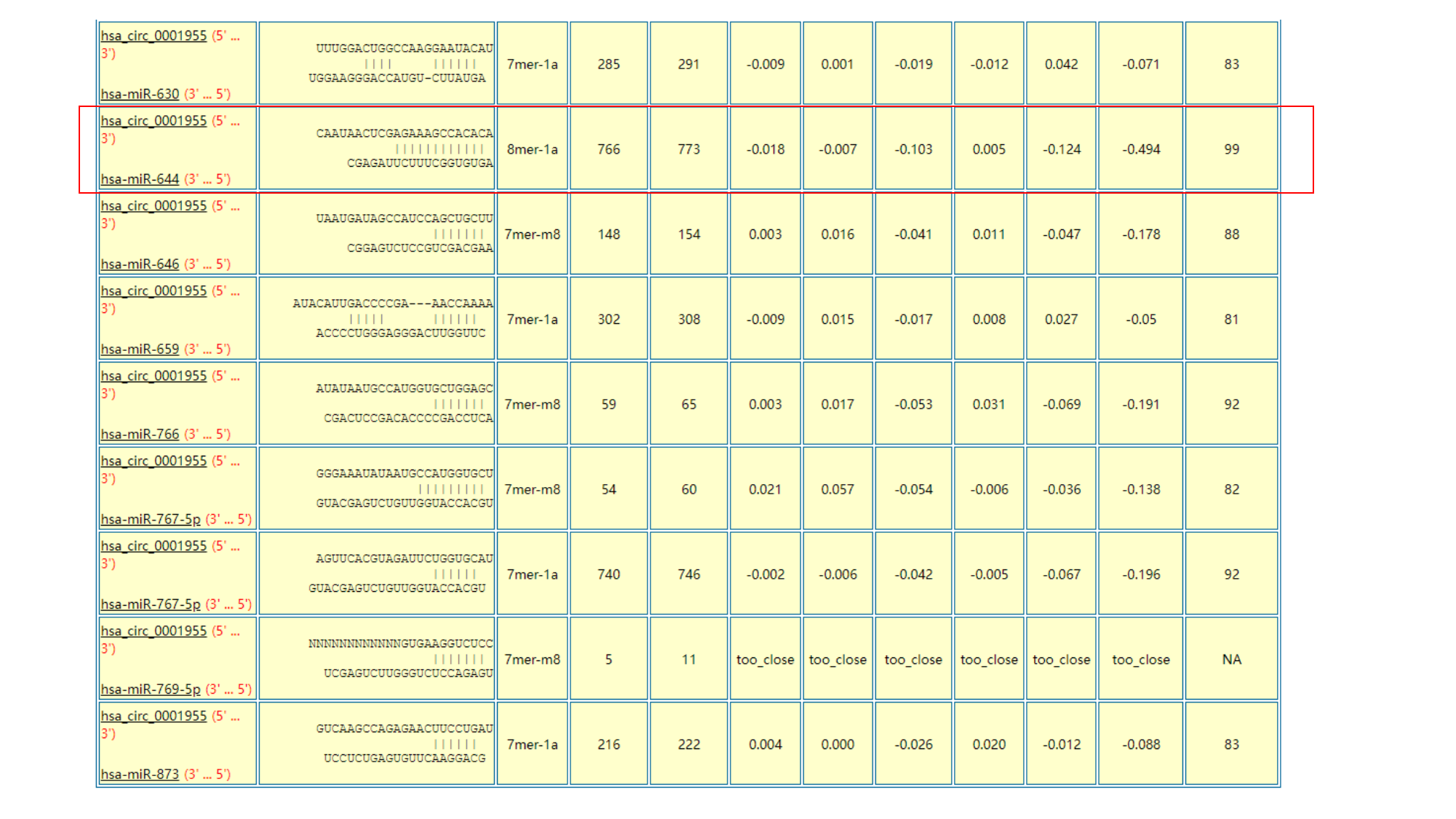

#

## Slide 5
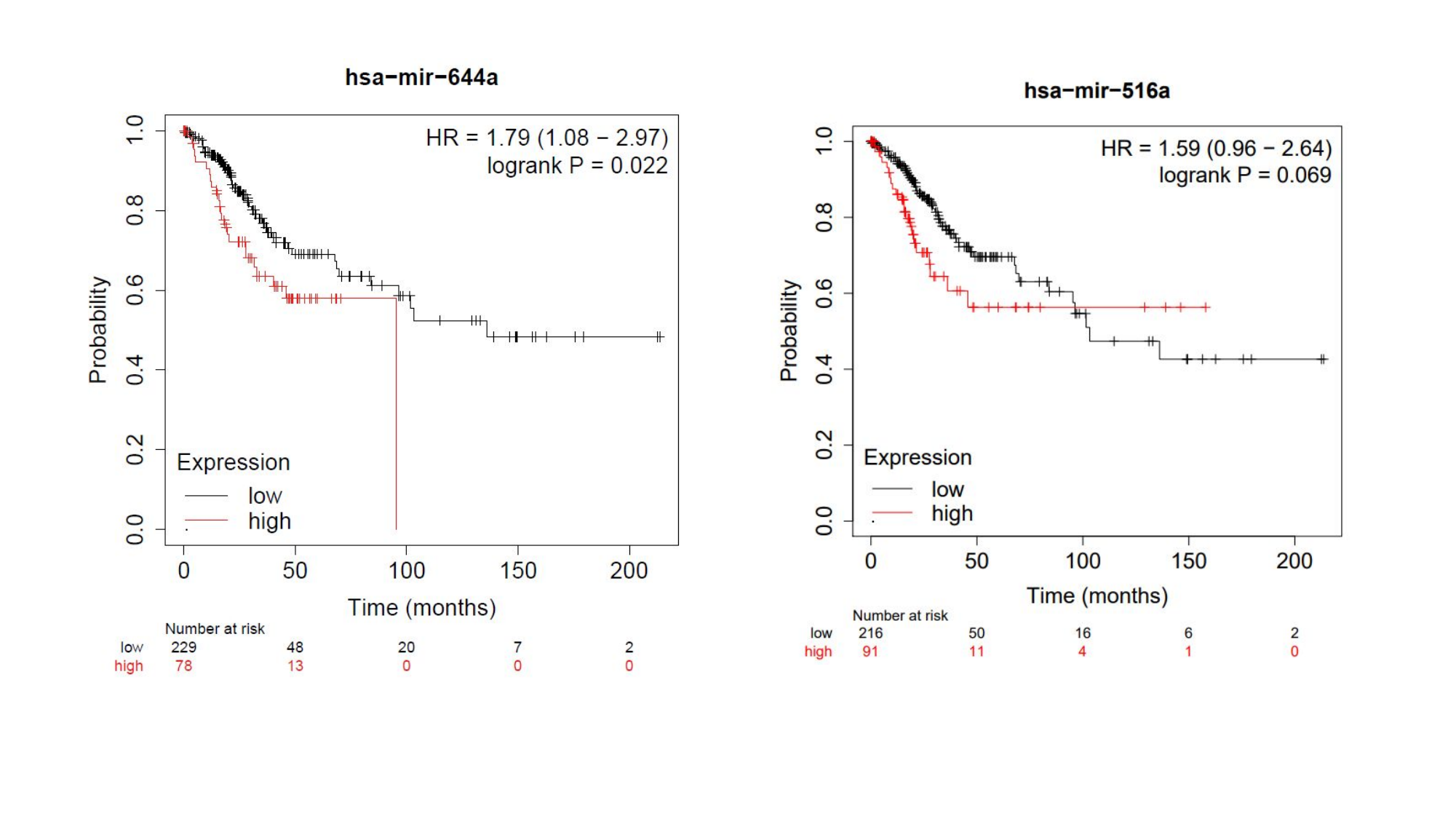

#

Supplement: Supplementary file 5 — Additional file 5: TargetScan database predicts potential target miRNAs of circ0001955 and Kaplan-Meier survival curve of overall survival based on TCGA database with CSCC according to the miR-644a and miR-516a expression. [file 12967_2023_4194_MOESM5_ESM.pptx]
